# Income and obesity in an urban poor community: a cross-sectional study

**DOI:** 10.12688/f1000research.22236.1

**Published:** 2020-03-03

**Authors:** Jo Ann Andoy-Galvan, Halyna Lugova, Sapna S. Patil, Yin How Wong, Gul M. Baloch, Adlina Suleiman, Rusli Nordin, Karuthan Chinna

**Affiliations:** 1 School of Medicine, Faculty of Health and Medical Sciences, Taylor's University, Subang Jaya, Selangor, 47500, Malaysia; 2 Faculty of Medicine and Defence Health, National Defence University of Malaysia, Sungai Besi, Kuala Lumpur, 57000, Malaysia

**Keywords:** obesity, prevalence, risk factors, socio-economic status, income

## Abstract

**Background**: Recent studies have shown that higher income is associated with a higher risk for subsequent obesity in low- and middle-income countries, while in high-income countries there is a reversal of the association – higher-income individuals have a lower risk of obesity. The concept of being able to afford to overeat is no longer a predictor of obesity in developed countries. In Malaysia, a trend has been observed that the prevalence of obesity increases with an increase in income among the low-income (B40) group. This trend, however, was not further investigated. Therefore, this study was performed to investigate the association of income and other sociodemographic factors with obesity among residents within the B40 income group in an urban community.

**Methods: **This cross-sectional study used a systematic sampling technique to recruit participants residing in a Program Perumahan Rakyat (PPR), Kuala Lumpur, Malaysia. The sociodemographic characteristics were investigated through face-to-face interviews. Weight and height were measured, and body mass index (BMI) was calculated and coded as underweight, normal, overweight and obese according to the cut-off points for the Asian population. A chi-squared test was used to compare the prevalence of obesity in this study with the national prevalence. A generalized linear model was introduced to identify BMI predictors.

**Results: **Among the 341 participants, 25 (7.3%) were underweight, 94 (27.6%) had normal weight, 87 (25.5%) were overweight, and 135 (39.6%) were obese. The proportion of obese adults (45.8%) was significantly higher than the national prevalence of 30.6% (p<0.001). Among all the tested variables, only income was significantly associated with BMI (p=0.046).

**Conclusion:** The proportion of obesity in this urban poor community was higher compared with the national average. BMI increased as the average monthly household income decreased.

## Introduction

Obesity prevalence has tripled since the 1970s. Currently, it has reached epidemic proportions throughout the globe and become a significant cause of morbidity and mortality ( WHO, 2020). It is a major risk factor in the development of the leading causes of global deaths, such as diabetes, cardiovascular diseases and cancer ( Al-Goblan *et al.*, 2014; De Pergola & Silvestris, 2013; Hubert *et al.*, 1983).

Malaysia is the most obese country among the Southeast Asian nations ( Ng *et al.*, 2014). Two out of three Malaysians are overweight or obese ( Aris *et al.*, 2015). Large-scale obesity studies in the country showed a two-fold increase in the prevalence of people categorised as overweight, from 16.6% to 30%, and a four-fold increase in obesity prevalence, from 4.5% to 17.7%, in the last two decades ( Aris *et al.*, 2015; Lin *et al.*, 2006). The main driver of obesity is an imbalance between energy consumption and expenditure ( Romieu *et al.*, 2017). However, our choices of eating and physical exercise are influenced by multiple factors and the concept of an obesogenic environment has been a topic of interest lately, that is, an environment that increases our risk of gaining weight and becoming obese ( Lipek *et al.*, 2015). Recent studies have shown complexity in the association of income and obesity: a higher income is associated with a higher risk of subsequent obesity in low- and middle income countries, but in high-income countries the reverse is observed, where those with a higher income are less likely to be obese ( McLaren, 2007). The concept that richer people can afford to overeat may no longer be true among well-developed nations. The reversal hypothesis was systematically tested using individual- and aggregate-level data for 67 nations representing all regions of the world and findings were consistent with individual studies in different countries: the influence of socioeconomic status on obesity shifts from positive to negative with national income ( Pampel *et al.*, 2012).

According to income classification in Malaysia, the income groups B40, M40 and T20 represent the lowest 40%, middle 40%, and highest 20% of incomes, respectively, among the country’s population. A national large-scale obesity study in 2015 revealed a positive trend among the low-income (B40) group but a mixed trend among the middle- and high-income groups ( Aris *et al.,* 2015). Also worth noting is the high prevalence rate of obesity in states with the highest (Putrajaya) and lowest urbanization (Perlis) levels ( Aris *et al.,* 2015; Department of Statistics, Malaysia, 2010). Nevertheless, the observed trend may not be universally applicable across heterogenous rural and urban community settings, where pockets of poverty are often found.

The inconsistencies with regards to the positive and negative associations of income with obesity might be due to a lack of studies among poor communities. These communities might differ in obesity prevalence along with its associated factors, and there are no current studies that explore this phenomenon. Therefore, this study was performed to investigate the association of income and other sociodemographic factors associated with obesity among residents within the B40 income group in an urban community.

## Methods

### Study design and setting

This cross-sectional study was carried out among the residents of the low-cost high-rise flats of a Community Housing Program, or PPR ( *Program Perumahan Rakyat* in local language) in Kuala Lumpur. PPRs have been developed by the National Housing Department (Jabatan Perumahan Negara or JPN) since the 1998 in an effort to provide affordable housing to low socioeconomic groups ( Goh *et al.,* 2011). A group of 150 medical students were deployed to collect data from all the 21 floors of the two residential building blocks during weekends of February 2019 and August 2019.

### Participants

**Figure 1.**
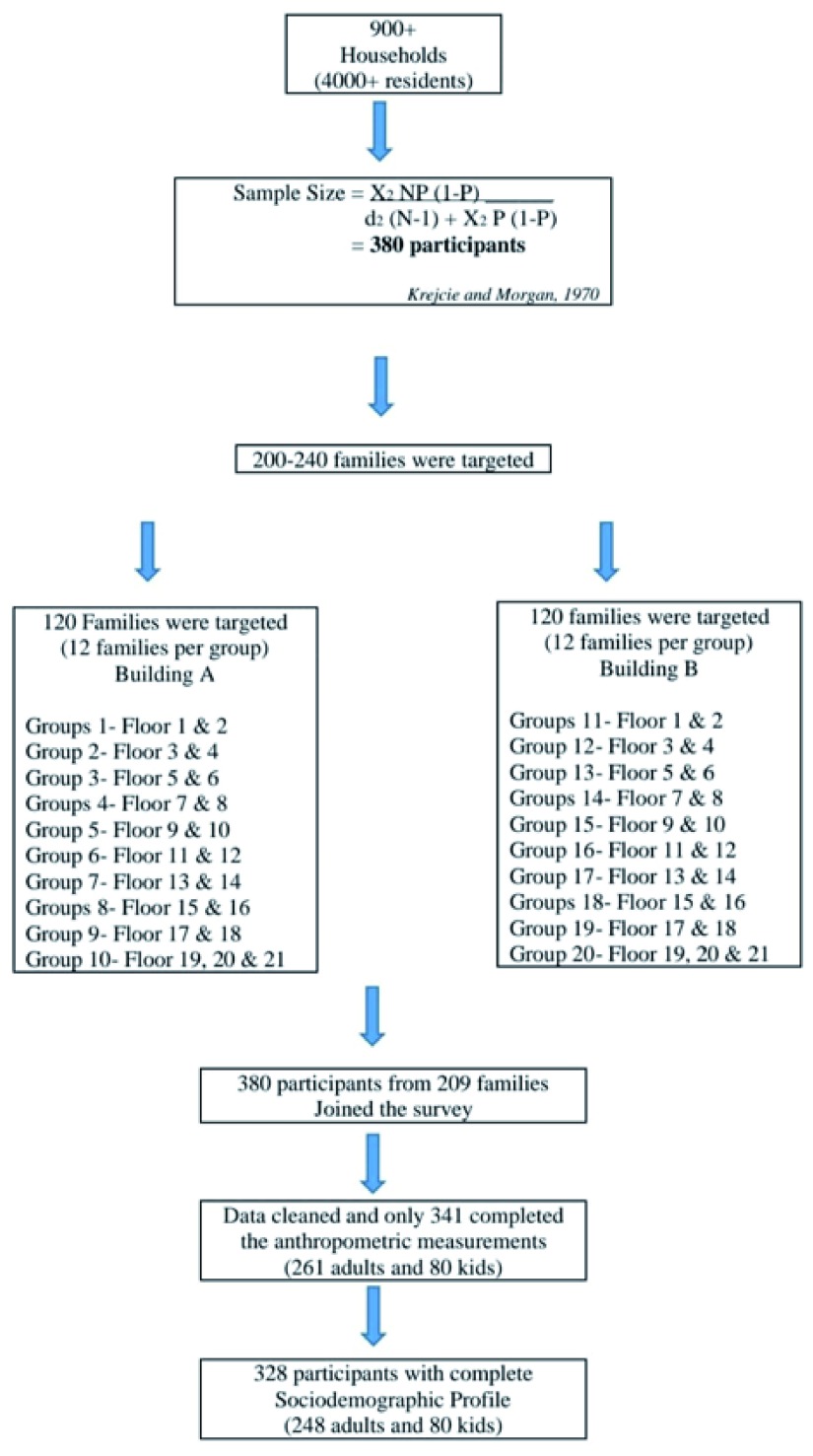
Participant flow chart

The sample size ( *n)* was calculated using the Krejcie and Morgan formula for prevalence studies of a known population ( Krejcie & Morgan, 1970). Prevalence used in the formula was the current prevalence of people who are overweight and obese for both genders, which is close to 50% ( Aris *et al.,* 2015). Out of 4229 residents, a total of 380 residents were expected to participate in the survey. Residents were approached at their home by data collectors using a systematic sampling procedure to ensure even representation of units from all the 21 floors. Data collectors were divided into 20 groups, each containing 7–8 collectors, 10 of which were assigned to building block A and 10 of which were assigned to building block B. Each group was assigned 2–3 floors of the 21-floor building block and were instructed to carry out systematic random sampling on their floors. If residents of one house did not consent, the data collectors moved to the next house. Within the selected units, all the residents were invited for an interview. The inclusion criteria included: (1) residents of the PPR; (2) Malaysian citizens; (3) aged =/>5 years old. Physically disabled/bedridden individuals were excluded ( Figure 1).

### Data collection

Sociodemographic data, including age, gender, race, educational level, marital status, household income and occupation, were collected by interviewing the residents using a questionnaire, and then their weight and height were measured to calculate their body mass index (BMI). Interviews were carried out by the data collectors; third year and fourth year medical students who had been trained to use the questionnaire. These data collectors had no prior interaction with the participants, but researchers regularly visit the community as part of ongoing research at our university. Interviews took place in the participants’ homes and lasted 30–45 minutes.

Body weight of the participants was measured in light clothing without footwear using Omron HBF-375 Body Composition Monitor digital weighing scale (Omron, Petaling Jaya, Malaysia). Height was measured without shoes using a measuring tape to the nearest 0.1 cm. BMI of each participant was calculated using a standard formula of weight (kg) divided by the square of the height (m ^2^) in kg/m ^2^.

### BMI categorization

The participants were categorized based on the classification of the 2004 Malaysian Clinical Practice Guidelines (CPG) on Management of Obesity ( Ismail *et al.,* 2004) which has adopted the recommended cut off points for the Asian population ( Nishida *et al.*, 2004). Under these guidelines, BMI was classified into six categories: underweight (<18.50 kg/m ^2^), normal (18.50 – 22.99 kg/m ^2^), overweight (23.00 – 27.49 kg/m ^2^), obese I (27.50 – 34.99 kg/m ^2^), obese II (35.00 – 39.99) and obese III (>40 kg/m ^2^) ( Zainudin *et al.*, 2011). For this study, we combined the obese I, II and III into one category of “obese”. For comparison with the national data, the BMI was also categorized according to the WHO Classification. ( Aris *et al.,* 2015).

For children aged 5 to 17 years, the BMI-for-age percentile based on Center for Disease Control growth charts, which are gender-specific, was used to categorize BMI ( Kuczmarski *et al.,* 2002).

### Statistical analysis

Data were analysed using IBM Statistical Package for Social Sciences version 23 (IBM, New York, USA). Frequencies, means, standard deviations were used to summarize the findings. Chi square was used to compare the obesity prevalence of the sample group with the national prevalence. Household income was skewed to the right; hence, it was log transformed. All the predictor variables were tested using multivariate analysis to determine the association with BMI. We used an alpha level of 0.05 for all the statistical tests.

### Ethical statement

This study received Institutional Review Board approval from Taylor's University Center for Research Management (HEC 2019/058). The study was conducted according to the Declaration of Helsinki. Prior written informed consent was obtained from all participants for participation and publication of data.

## Results

There was a total of 380 participants, of which 341 completed anthropometric measurements. Of these, there was a total of 328 respondents, including 261 adults and 80 children (aged below 18 years old) from 209 household units, who provided full sociodemographic information ( Galvan, 2020). Table 1 shows the sociodemographic characteristics of the 328 participants. Among the 261 adult participants that completed anthropometric measurements, 29.1% and 45.6% were overweight and obese, respectively ( Table 2). Among the respondents aged below 18 years, the prevalence of an overweight and obese BMI was 13.8 % and 20%, respectively ( Table 3).

**Table 1.** Socio-demographic variables (N=328).

Variables	*n*	(%)
Age		
5–17	80	(24.4)
18–34	86	(26.2)
35–54	109	(33.2)
<55	53	(16.2)
Race		
Malay	197	(60.1)
Chinese	7	(2.1)
Indian	124	(37.8)
Gender		
Male	113	(34.5)
Female	215	(65.5)
Education		
None	39	(11.9)
Primary	79	(24.1)
Secondary	122	(37.2)
Tertiary	88	(26.8)
Occupation		
Under six years old	4	(1.2)
Student	90	(27.4)
Not working	120	(36.6)
Working	114	(34.8)
Marital status		
Single	136	(41.5)
Married	176	(53.7)
Widowed	12	(3.7)
Divorced	4	(1.2)
Monthly household income RM (mean ± SD)	1149.32	± 1059.83
Monthly household income (log transformed)	2.91	± 0.37
Height in meters (mean ± SD)	153.29	± 14.43
Weight in kg (mean ± SD)	60.97	± 20.57
BMI in kg/m ^2^ (mean ± SD): children	19.51	± 5.41
BMI in kg/m ^2^ (mean ± SD): adults	27.17	± 6.23

BMI, body mass index; RM, Malaysian ringgit.

**Table 2.** Distribution of BMI among adults according to BMI classification.

Categories	BMI ^a^	*n*	(%)	BMI ^b^	*n*	(%)
Underweight	<18.5 kg/m ^2^	15	(5.7)	<18.5 kg/m ^2^	15	(5.7)
Normal	18.5–22.9 kg/m ^2^	51	(19.5)	18.5–24.9 kg/m ^2^	86	(33.0)
Overweight	≥ 23.0 kg/m ^2^	76	(29.1)	≥ 25.0 kg/m ^2^	82	(31.4)
Obese	≥ 27.5 kg/m ^2^	119	(45.6)	≥ 29.9 kg/m ^2^	78	(29.9)

^a^CPG, Clinical Practice Guidelines in Malaysia ^b^WHO, World Health Organization. BMI, body mass index.

**Table 3.** Distribution of BMI among children (<18 years old) according to the BMI-for-age percentiles.

Category	BMI	*n*	(%)
Underweight	less than 5th percentile	10	(12.5)
Normal	5th to <85th percentile	43	(53.8)
Overweight	85th to <95th percentile	11	(13.8)
Obese	95th percentile or greater	16	(20.0)

BMI, body mass index.

### Comparison of the prevalence of obesity in this study and among general population in Malaysia

As shown in Table 4, the prevalence of obesity in our sample was significantly higher as compared to the average national prevalence by using both CPG classification of BMI (45.6% *vs.* 30.6%, p<0.001) and WHO classification of BMI (29.9% *vs.* 17,7%, p<0.001). The prevalence of obesity in this study was similar to that observed in the federal territory of Putrajaya (43%), which has the highest obesity prevalence in the country ( Aris *et al.*, 2015).

**Table 4.** Comparison of the prevalence of overweight and obese participants in study population with the average national prevalence.

BMI Category	Prevalence (%) CPG Classification		X ^2^	p value	Prevalence (%) WHO Classification		X ^2^	p value
	National sample	Study sample			National sample	Study sample		
Overweight	33.40	29.11	2.15	0.143	30.0	31.42	0.250	0.617
Obese	30.60	45.60	27.63	**<0.001**	17.70	29.86	26.60	**<0.001**

BMI, body mass index; CPG, Clinical Practice Guidelines in Malaysia; WHO, World Health Organization.

### Evaluation of risk factors for obesity among the adults in the community

A generalized linear model was used to test the association between the predictor variables and BMI. As shown in Table 5, among all the tested variables, gender and income were significantly associated with BMI. Participants with a higher income had a lower BMI (b -1.86; p value 0.046). Males had a lower BMI than females (b-1.475; p value 0.037).

**Table 5.** Association of sociodemographic variables with BMI (N=328).

Variables	*Mean (Std. deviation)*	*P value*
Age		0.44
<18	19.51 (5.41)	
18–34	25.71 (6.93)	
35–54	28.72 (6.05)	
>55	26.51 (4.59)	
Race		0.284
Malay	25.44 (6.80)	
Chinese	24.09 (2.84)	
Indian	25.22 (7.19)	
Gender		0.037
Male	23.49 (6.12)	
Female	26.29 (7.07)	
Education		0.390
None	23.76 (6.84)	
Primary	22.73 (6.50)	
Secondary	26.65 (6.84)	
Tertiary	26.52 (6.59)	
Occupation		0.413
Student	20.10 (5.60)	
Not working	27.53 (6.63)	
Working	27.47 (5.58)	
Under six	16.53 (6.88)	
Marital Status		0.287
Single	21.66 (6.53)	
Married	27.96 (5.76)	
Widowed	27.08 (7.44)	
Divorced	28.67 (7.73)	
Monthly household income	-1.86 * (0.9354)	0.046

b (SE)* BMI, body mass index.

## Discussion

The obesity prevalence in this study was significantly higher compared to the national prevalence ( Aris *et al.*, 2015). Contrary to the results from the national survey ( Aris *et al.*, 2015), income was negatively associated with BMI after controlling for other variables in this low-income (B40) population group; in other words, the individuals with a higher income had a lower BMI. The negative association was similar to that of high-income countries, where a socioeconomically developed environment may possibly contribute to the reversal ( Pampel *et al.*, 2012). In contrast to the built environment in rural areas, families with a higher income among this community may be able to afford accessible healthy food choices along with various available weight loss programs, walkable entertainment centres, shopping malls and healthcare facilities ( Ellis *et al.,* 2016); thus, they are less obese as compared to the rest of the community. This supportive environment decreases the risk of obesity for urban dwellers, while the concept of being able to afford to overeat may still be true among wealthy rural dwellers ( Creatore *et al.,* 2016). Our results confirm the arguments discussed in the review of 2,009 population-based studies done by the NCD Risk Factor Collaboration ( Bixby *et al.,* 2019). The researchers found that more than 55% of the rise in global obesity was from rural areas. Their findings negate the assumptions that urbanization was the main driver of the obesity epidemic. Assumptions of sedentary lifestyles with access to processed food in urban places, and the general preconceived notion of manual labor and healthy eating from one’s own garden among rural dwellers may not be true. Automation has influenced our agricultural activities and distribution or shipment of processed food to rural areas. ( Bixby *et al.,* 2019) In the national survey done by Aris *et al.* (2015), both the states with highest and lowest urbanization levels had high prevalence rates of obesity. Putrajaya, with 100% urbanization, reported a 43% rate of obesity and Perlis, with 51.4% urbanization, reported a rate of 36% rate of obesity, ranking 2 ^nd^ together with the state of Melaka. This report contradicts the general idea that obesity applies to urban communities and malnutrition to rural communities. In our report, this urban poor community has a greater prevalence of obesity compared to the general population but the distribution of obesity among them was skewed towards those with a low income. The driver could possibly be that the advantages of the available supportive environment, such as gyms and healthy food choices, in the urban-built environment are missed by those with a lower income.

## Conclusion

The obesity rate in this urban poor community is high. The monthly household income is negatively associated with obesity; people with a higher income have a lower BMI.

To understand further the complexity of the relationship between income and obesity, we recommend further investigations involving more than one PPR community. Determining monthly food and health expenses of the families will increase the precision of information on the household income level. Additionally, a wider study could compare trends between urban and rural poor communities.

## Limitations

Our findings are limited to one community, and the cross-sectional nature of this study does not permit any temporal relationships to be deduced. Monthly household income disclosed to the interviewers may not reflect the actual figures; the residents in this community are recipients of the government housing project and therefore represent the poorest 40% of the population and should receive a salary of ~3,000 ringgit or below. However, salaries higher than 3,000 ringgit were reported in this study.

## Data availability

### Underlying data

Harvard Dataverse: Income and obesity in an urban poor community: a cross-sectional study. https://doi.org/10.7910/DVN/XRO2PI ( Galvan, 2020)

This project contains the following underlying data:

- Adults_22236F.tab (BMI data for adults in the study)
- Children_22236.tab (demographic and BMI data for children in the study)
- Combined_Data_22236F.ods (demographic and BMI data for all participants in the study)

Data are available under the terms of the Creative Commons Zero "No rights reserved" data waiver (CC0 1.0 Public domain dedication).

Household income data are not included in the dataset but will be available upon request from the corresponding author. This is to protect the confidentiality of the income received by the residents living in a housing project intended for the poorest families in the country. There are numerous determinants of salary, which lead to diverse income figures among the residents and this should not be misinterpreted by taking the information on the income separately. Researchers who would like to acquire the data for the purpose of statistics may write to the corresponding author ( JoAnnAndoy.Galvan@taylors.edu.my) stating reasons for the request.
