# Supplementary material for: Income and obesity in an urban poor community: a cross-sectional study
Source: F1000Res. 2020 Mar 3;9:160. [Version 1] doi: 10.12688/f1000research.22236.1 (PMC7194455; doi:10.12688/f1000research.22236.1)
Supplement: Peer review history

# Peer review: Reviewer response for version 1

**DOI:** 10.5256/f1000research.24525.r62102

**Published:** 2020-04-23

**Authors:** Khalid Abdul Kadir

**Affiliations:** 1 School of Medicine and Health Sciences, Monash University Malaysia, Bandar Sunway, Malaysia

This is a cross sectional study of the prevalence obesity and overweight amongst the lower 40% of a Malaysian population living in an urban government housing scheme. The methodology used was very appropriate a properly calculated sample size for the population, and using systemic sampling procedure with face to face interviews etc.

 The criteria used for overweight and obesity was also appropriate for the Asian/Malaysian population

 The data obtained included socio demographic data including income

 There was no data about the diet or food calorie intake nor of energy expenditure ie physical work, exercise etc 

 The data obtained was interesting and novel in that it showed increased prevalence of obesity/overweight in the lower income group but the prevalence is less in this group if the income is higher.

 The prevalence is then compared to a recent nationwide prevalence survey by Aris et al. and showed higher prevalence compared to national average.

 The findings would have been improved if they had looked at the racial group involved compared to the national data, and to include the nutritional and dietary habits

 Overall a short interesting paper.

Is the work clearly and accurately presented and does it cite the current literature?

Yes

If applicable, is the statistical analysis and its interpretation appropriate?

Partly

Are all the source data underlying the results available to ensure full reproducibility?

Yes

Is the study design appropriate and is the work technically sound?

Yes

Are the conclusions drawn adequately supported by the results?

Yes

Are sufficient details of methods and analysis provided to allow replication by others?

Yes

Reviewer Expertise:

Diabetes, obesity and metabolic diseases, epidemiology of diabetes and metabolic diseases, clinical trials on diabetes, animal studies on diabetes and metabolic syndrome

I confirm that I have read this submission and believe that I have an appropriate level of expertise to confirm that it is of an acceptable scientific standard.

# Peer review: Reviewer response for version 1

**DOI:** 10.5256/f1000research.24525.r61436

**Published:** 2020-03-31

**Authors:** Gajanan Dattatray Velhal

**Affiliations:** 1 Department of Community Medicine, eth G S Medical College & KEM Hospita Mumbai, Mumbai, Maharashtra, India

Contrary to the existing belief that Obesity is more common among the people belonging to higher socioeconomic status, present study reveals that prevalence of obesity is seen more among individuals with low income categories, whose economic status is improving. This clearly indicates the need to focus on the selected category of people to influence overall prevalence of obesity in the community. Reasons to exclude physically handicapped and bed ridden patients is not clear. It would have been better to give elaborate description on employment of systematic random sampling technique for selection of study subjects.

Is the work clearly and accurately presented and does it cite the current literature?

Yes

If applicable, is the statistical analysis and its interpretation appropriate?

Yes

Are all the source data underlying the results available to ensure full reproducibility?

Yes

Is the study design appropriate and is the work technically sound?

Yes

Are the conclusions drawn adequately supported by the results?

Yes

Are sufficient details of methods and analysis provided to allow replication by others?

Yes

Reviewer Expertise:

Nutrition, Epidemiology of Communicable and Non communicable diseases, Epidemiological studies

I confirm that I have read this submission and believe that I have an appropriate level of expertise to confirm that it is of an acceptable scientific standard.
